# Association of *SNP rs.2414096 CYP19* gene with polycystic ovarian syndrome in Iranian women

**Published:** 2017-08

**Authors:** Anahita Mehdizadeh, Seyed Mehdi Kalantar, Mohammad Hassan Sheikhha, Bibi Shahnaz Aali, Azam Ghanei

**Affiliations:** 1 *Biotechnology Research Center, International Campus, Shahid Sadoughi University of Medical Sciences, Yazd, Iran.*; 2 *Research and Clinical Center for Infertility, Yazd Reproductive Sciences Institute, Shahid Sadoughi University of Medical Sciences, Yazd, Iran.*; 3 *Physiology Research Center, Department of Obstetrics and Gynecology, Afzalipour Hospital, Kerman University of Medical Sciences, Kerman, Iran.*; 4 *Department of Internal Medicine, Shahid Sadoughi Hospital, Shahid Sadoughi University of Medical Sciences, Yazd, Iran.*

**Keywords:** Polycystic ovarian syndrome, CYP19, Single nucleotide polymorphism

## Abstract

**Background:**

Genetic factors are believed to play an important role in the etiology of polycystic ovarian syndrome (PCOS) which is the most common endocrinological disorder of women in their reproductive age. Androgen metabolism is impaired in PCOS and, thus, *CYP19* gene which is involved in this pathway can be a candidate gene. Previous studies have shown a relationship between single nucleotide polymorphism (SNP) of *CYP19* in hyperandrogenism and PCOS in some racial groups.

**Objective:**

This study was designed to elucidate the role of *CYP19* gene in PCOS in Iran.

**Materials and Methods:**

In this case-control study, 70 PCOS women and 70 non-PCOS women as normal control were selected. Following the informed consent, 5 ml blood was taken from individuals and subsequently, genomic DNA was extracted by salting out method. Furthermore, a set of polymerase chain reaction restriction fragment length polymorphism (PCR-RFLP) was carried out using specific primers for SNP rs.2414096 followed by enzyme digestion, with HSP92II.

**Results:**

Genotype frequencies of SNP rs. 2414096 in PCOS women were as follows: AA (14.4%), AG (44.3%), and GG (41.4%) while in normal group, genotypes were 24.3%, 52.8%, and 22.9%, respectively. Allele frequencies in PCOS group were 49.3% for A and 50.7% for G, whereas normal group had a different percentage of A (36.4%) and G (63.6%). The calculations for both genotypic and allelic frequencies showed statistical significance difference.

**Conclusion:**

Variants of SNP rs. 2414096 in *CYP19* could play a role in the development of PCOS in Iranian women.

## Introduction

Polycystic ovarian syndrome (PCOS) is the most common endocrinopathy in women with the prevalence of 5-10% worldwide (1). The main characteristics of this syndrome are clinical and/or biochemical hyperandrogenism, ovulatory dysfunction and polycystic ovaries in ultrasound (2). Although little is known about the etiology of PCOS, accumulative evidence such as familial aggregation of PCOS individuals suggest a strong involvement of genetic factors in this area (3). Furthermore, heterogeneous clinical manifestations which are impacted by ethnicity are the main proofs for environmental factors to be involved in the origin of PCOS (4). Excess production of ovarian androgens is one of the major physiopathological features of PCOS. Previous studies revealed several genes such as *CYP11A1*, *CYP17*, *HSD17B6*, *INSR* and *CYP19* which encode essential enzymes in androgen biosynthesis pathways (2, 5-8). However, there were discrepancies in the results of researches regarding the association of these genes.

*CYP19* resides on chromosome 15 (15q21.1), spans 123Kb and contains 10 promoters in a large regulatory region (9, 10). *CYP19* gene encodes cytochrome P450 aromatase, the key enzyme which catalyses the final step of conversion of androgens (C19 steroids, testosterone and androstenedione) to estrogens (C18 steroids, estradiol and estrone) in gonadal and extragonadal tissues (11-14). Variations in *CYP19* have potential impacts on human health either by influencing the amount of testosterone available for androgen receptor binding or estrogen available for receptor binding (15). Increase in androgen to estrogen ratio is a consequence of variation in *CYP19* and may associate with hyperandrogenic phenotype in PCOS patients (16).

Previous investigations reported an association between some single nucleotide polymorphisms (SNPs) and variants of *CYP19* and different levels of androgen concentration in women in different ethnical and racial groups (15). It has been elucidated that the SNP 2414096 in *CYP19* gene is associated with hyperandrogenism. Results of a case-controlled study in China revealed the association of SNP 2414096 with altered regulation of aromatase enzyme and possibility of PCOS (17). 

Since to our knowledge, there is no research about the genotype distribution of SNP rs.2414096 in *CYP19* gene in Iranian population (PCOS vs. normal), therefore the following study was designed to examine the possible role of *CYP19* gene variants in the development of PCOS.

## Materials and methods

**Subjects**

In this case-control study, a total number of 140 Iranian women referred to the Department of Obstetrics and Gynecology, Afzalipour Hospital, Kerman University of Medical Sciences from September 2013-2014 consisted our study population, including 70 PCOS affected women (18-40 yr old) and 70 aged matched non-PCOS control women with normal menstrual cycles (between 28-32 days), lean (nonobese), and lacking the signs of hirsutism, severe acne, hair loss and insulin resistance. Individuals with PCOS were recruited according to Rotterdam 2003 criteria which require the presence of at least two criteria: clinical and/or biochemical hyperandrogenism, ovulatory dysfunction and polycystic ovary morphology with exclusion of hyperthyroidism, hyperprolactinemia, and other hyperandrogenism causes such as congenital adrenal hyperplasia, androgen-secreting tumors and Cushing’s syndrome (18). 

**Genotyping analysis of SNP rs.2414096**

Genomic DNA was extracted from leukocytes of peripheral blood samples by standard salting out method. Genotyping of SNP 2414096 of *CYP19* gene was performed by polymerase chain reaction (PCR). The sequence for forwarding primer was 5’-TCT GGAAACTTTTGGTTTGAGTG-3’ and reverse primer was 5’-GATTTAGCTTTAGAGCCTTTT CTTACA-3’. PCR reactions were performed in a Conversion (TCY48) thermocycler in a total volume of 30 μL containing 100 ng of template DNA, 10 μL Master mix which was composed of Taq DNA polymerase, MgCl_2_, PCR buffer and dNTPs, 1 μL of each forward and reverse primers and 13 μL sterile water. PCR reactions consisted of 35 cycles of 30 sec of denaturation at 95^o^C, 30 sec of annealing at 56.8^o^C and 30 sec of extension at 72^o^C. The initial step of denaturation was 5 min at 95^o^C and final extension step was programmed for 5 min at 72^o^C.

**Figure 1:**
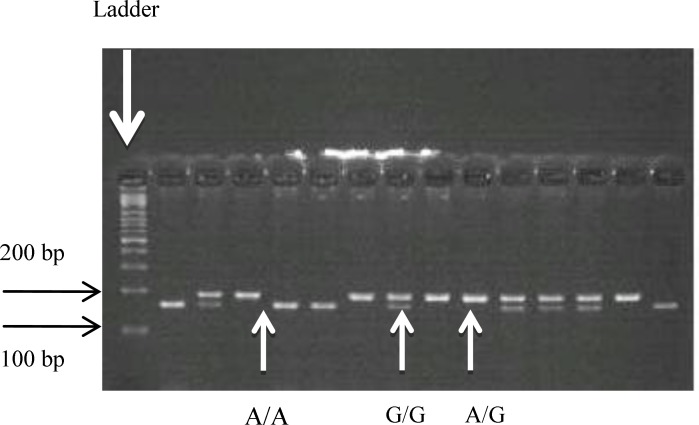
Result of the enzyme digestion with HSP92II.

The subsequent enzyme digestion, was done with HSP92II at 37^o^C for 1-4 hr. A single 189 bp, band indicates the wild type “G” homozygote. Two bands of 161 bp and 28bp show “A” homozygote genotype and a combination of 189 bp, 161 bp, and 28 bp bands correspond to “AG” heterozygote. The enzyme digested products were separated on 2% agarose gel and stained with ethidium bromide and visualized under UV (Figure 1).

**Ethical consideration**

The study protocol was approved by the ethical committee of International Campus of Shahid Sadoughi University of Medical Sciences and the informed consent was taken from all the participants of the study. 

**Statistical analysis**

Comparison of the distribution of *CYP19* gene genotype was performed by Fisher’s Exact Test. This analysis was done using the GraphPad Prism5 version 5.01 software. p<0.05 was considered as statistically significant.

## Results

In a total number of study participants (140), the general distribution of different genotypes was 0.193 for AA, 0.486 for AG and 0.321 for GG. The PCOS group showed the following genotypic distribution (0.143 AA, 0.443 AG and 0.414 GG), which were significantly different from normal controls (0.243, 0.528 and 0.229 respectively) (p=0.047) (Table I). The frequency distribution of *CYP19* rs.2414096 alleles in PCOS and controls were 0.36 for A and 0.64 for G in comparison to 0.51 and 0.49 in controls for A and G respectively (p=0.02) (OR=1.8 95% CI=1.113-2.896).

**Table I:** Frequency distribution of different genotypes and alleles of SNP rs.2414096 CYP19 gene in control and PCOS groups

**SNP rs.2414096**	**PCOS**	**Controls**	**p-value**
AA genotype	14.3	24.3	0.047
AG genotype	44.3	52.8	
GG genotype	41.4	22.9	
A allele	36	51	0.02
G allele	64	49	

All data presented as %

Alleles: Fisher’s exact test applied

OR: 0.556

Genotypes: Fisher’s exact test applied

PCOS: polycystic ovarian syndrome

**Figure 2:**
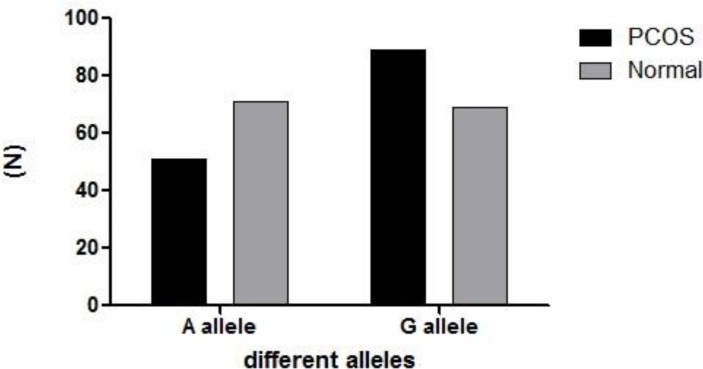
The allelic distribution of SNP rs.2414096.

**Figure 3:**
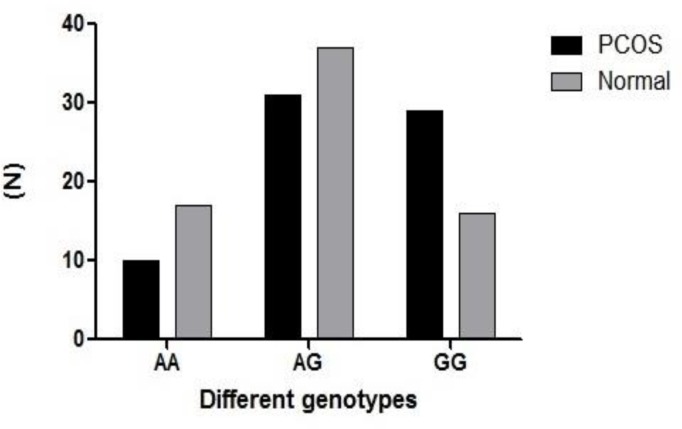
The genotypic distribution of SNP rs.2414096

## Discussion

Although PCOS is characterized as a heterogeneous disorder, accumulative evidence based on concordance in twins and aggregation of familial cases support its genetic base. Considering the lack of universally accepted criteria for PCOS, studies were failed to nominate a single pathogenic gene for triggering PCOS, though previous investigations revealed information on the possible effect of SNPs of *CYP19* gene. The present study examined the possible pathogenic role of *CYP19*, particularly SNP rs.2414096 and whether different genetic variants of rs.2414096 can be related to PCOS predisposition. Our results revealed that there is a significant difference in genotypic and allelic frequencies for SNP rs.2414096 between the case (PCOS) and non-PCOS control groups. 

The statistical results of the distribution of genotypes indicate that individuals with GG and AG genotypes are at higher risk for PCOS when compared to AA genotype groups, whereas in control group, carriers of AA and AG consists the majority group. Consequently, “A” allele in PCOS affected individuals was less frequent than the control group. Therefore carriers of allele “G” are more susceptible to developing PCOS. As SNP rs.2414096 of *CYP19* gene is located in an intron, different forms of it can affect regulatory sequences, therefore the rs.2414096, “A” allele could be associated with normal function of different hormones which can protect the ovaries from developing PCOS, while a decline in the activity of these hormones could be linked to the “G” allele with further ovarian hyperandrogenism and subsequent PCOS (17). As it was suggested before, more studies in these regards are needed to measure the level of different hormones in individuals with different alleles.

As examples of related studies, we can mention the study performed by Jin *et al* in 2009 who concluded “A” allele is related to the activity of aromatase and the normal conversion of androgens to estrogens (17). They also found that estradiol to testosterone ratio in AA genotypic group of PCOS was markedly higher compared to the other two groups (AG and GG) which propose that activity of aromatase was augmented in AA genotypic group.

In another study, Petry *et al* reported the higher concentration of testosterone in allele “A” in precocious pubarche girls who were susceptible to develop PCOS, while in our results the incidence of PCOS was higher in individuals with “G” allele (2). The possible reason for the discrepancy might be firstly in the chosen criteria for selecting PCOS individuals and precocious pubarche girls. While the girls who participated in Petry *et al* study aged 9.8-10.9 yr at assessment, our participants were 18-40 yr at the time of assessment, and secondly, there is a difference in the distribution of A/G alleles between Middle Eastern and European populations (2).

Moreover in a study by Yu and colleagues in 2013, aimed to examine the methylation status of the *CYP19A1* promoter in Chinese PCOS and control, results revealed a frequent repression of *CYP19*A1 in PCOS ovaries as a consequence of promoter hypermethylation (19). Xu *et al* showed that PCOS patients had a higher frequency of (TT/TA) alleles in intron 4 of *CYP19* gene, albeit they concluded it may not affect the risk of PCOS (20). Also, TC genotype of SNP rs.2470152 in *CYP19* seems to inhibit aromatase activity and leads to hyperandrogenism in PCOS patients (21). Although the aforementioned researches were done on the different region (promoter) and SNPs of *CYP19* gene their results are in agreement with our results based on the fact that *CYP19* gene might be correlated with PCOS predisposition.

Finally, as PCOS is established to be a heterogeneous complex genetic disorder and is the most common endocrine disorder in women of reproductive age with molecular basis the study of different gene mutations such as the bone morphogenetic protein-15 gene which was studied by the author is important in the management of the disease (22, 23). The present study is the first one which reports the association of rs.2414096 in *CYP19* gene with PCOS in Iranian population.

## Conclusion

The results of this study could be an indication that there is a possibility of an association between SNP rs.2414096 in *CYP19* gene and the development of PCOS. More studies on different populations are needed to confirm this finding.

## Note

***This article extracted from M.Sc. thesis. (Anahita Mehdizadeh)***
